# Measuring vaccine effectiveness against persistent HPV infections: a comparison of different statistical approaches

**DOI:** 10.1186/s12879-020-05083-7

**Published:** 2020-07-08

**Authors:** R. Donken, J. Hoes, M. J. Knol, G. S. Ogilvie, S. Dobson, A. J. King, J. Singer, P. J. Woestenberg, J. A. Bogaards, C. J. L. M. Meijer, H. E. de Melker

**Affiliations:** 1 Vaccine Evaluation Center, BC Children’s Hospital Research, University of British Columbia, 950 West 28th Avenue, Vancouver, BC V5Z 4H4 Canada; 2 Women’s Health Research Institute, BC Women’s Hospital and Health Centre, Vancouver, BC Canada; 3 School of Population and Public Health, University of British Columbia, Vancouver, BC Canada; 4 Center for Infectious Disease Control, National Institute for Public Health and the Environment, Bilthoven, The Netherlands; 5 Care and Public Health Research Institute, Maastricht University Medical Center, Maastricht, the Netherlands; 6 Department of Pathology, VU University Medical Center, Amsterdam, the Netherlands

**Keywords:** Human papillomavirus, HPV, Persistent infection, HPV vaccination, Follow-up

## Abstract

**Background:**

Persistent high-risk human papillomavirus (HPV) infection is endorsed by the World Health Organization as an intermediate endpoint for evaluating HPV vaccine effectiveness/efficacy. There are different approaches to estimate the vaccine effectiveness/efficacy against persistent HPV infections.

**Methods:**

We performed a systematic literature search in Pubmed to identify statistical approaches that have been used to estimate the vaccine effectiveness/efficacy against persistent HPV infections. We applied these methods to data of a longitudinal observational study to assess their performance and compare the obtained vaccine effectiveness (VE) estimates.

**Results:**

Our literature search identified four approaches: the conditional exact test for comparing two independent Poisson rates using a binomial distribution, Generalized Estimating Equations for Poisson regression, Prentice Williams and Peterson total time (PWP-TT) and Cox proportional hazards regression. These approaches differ regarding underlying assumptions and provide different effect measures. However, they provided similar effectiveness estimates against HPV16/18 and HPV31/33/45 persistent infections in a cohort of young women eligible for routine HPV vaccination (range VE 93.7–95.1% and 60.4–67.7%, respectively) and seemed robust to violations of underlying assumptions.

**Conclusions:**

As the rate of subsequent infections increased in our observational cohort, we recommend PWP-TT as the optimal approach to estimate the vaccine effectiveness against persistent HPV infections in young women. Confirmation of our findings should be undertaken by applying these methods after longer follow-up in our study, as well as in different populations.

## Background

More than 30 types of the human papillomavirus (HPV) can infect the genital tract. Based on their oncogenic potential for cervical cancer, HPV types are divided into low- and high-risk (hrHPV) types. The majority of HPV infections are cleared by the immune system. However, remaining infections can persist within cells and progress to (pre)-cancerous lesions [1]. A persistent infection with HPV is the necessary cause for the development of cervical cancer. Beyond its role as etiological agent of cervical cancer, hrHPV is associated with other anogenital and oropharyngeal cancers in men and women [2]. Since 2006, three prophylactic vaccines have been licensed, and many countries have implemented HPV vaccination programs [3]. While these vaccines offer protection against two, four, or nine HPV types, all protect against hrHPV types 16 and 18. For the bivalent vaccine, cross-protection has been shown against additional types (HPV31, 33, 45) which are not included in the vaccine [4].

Given its role in the pathogenesis, persistent hrHPV infection is endorsed by the World Health Organization (WHO) as an intermediate endpoint for estimating HPV vaccine effectiveness/efficacy in cervical and anal cancer among 16–26 year olds [5]. In general, persistence is defined as presence of the same HPV type in consecutive measurements [6]. The use of persistent infections as an outcome for vaccine effectiveness/efficacy is more convenient than pre-cancerous lesion (e.g. cervical intraepithelial neoplasia), however it comes with several challenges. Besides uncertainties in the natural history of HPV infections, with possible viral latency and natural immunity after infection [7], difficulties in measuring vaccine effectiveness/efficacy might arise from longitudinal study designs with loss to follow-up and missing observations. In addition, clustered data can result from the possibility of infections with multiple HPV types (at once) and/or having recurrent detection (reinfection or reactivation) after a negative measurement. Additionally, the risk for recurrent detection might be higher than developing a first-time infection [8]. Another challenge is that rates of infection over time in young vaccinated cohorts might vary due to increasing sexual behavior in this age group [9]. This varying infection rate might influence which statistical approach is optimal for estimating vaccine efficacy/effectiveness in observational cohort studies.

In this paper, we identify and examine different approaches to estimate the vaccine effectiveness (VE) against persistent HPV infections from the literature, and determine whether the statistical assumptions of these approaches hold within data from an observational cohort study. Furthermore, we examine whether a violation of these statistical assumptions leads to bias in the estimation of the VE.

## Methods

### Literature search

A systematic literature search with no indicated start date till May 15, 2019 was performed in PubMed (detailed search strategy is in Additional file 1), to obtain insight into various methods used to estimate the vaccine effectiveness/efficacy against persistent HPV infections. Although vaccine efficacy and effectiveness vary in the conditions under which they are obtained, they both aim to measure the proportionate reduction in disease burden. Vaccine efficacy is studied under controlled circumstances, for example in a randomized controlled trial, while vaccine effectiveness is estimated from studies conducted under field circumstances [10]. Calculations of efficacy and effectiveness are comparable, especially in situations where the vaccine effectiveness aims to measure the direct effects by comparing the risk in vaccinated and unvaccinated participants [11]. Given our focus on observational studies, we will only use the abbreviation VE when vaccine effectiveness is described.

Papers were screened based on predefined inclusion criteria. Inclusion criteria covered original research papers estimating vaccine efficacy or effectiveness against persistent HPV infections (i.e. comparing different groups) for any prophylactic HPV vaccine, written in Dutch or English language. Data were extracted using a standardized data extraction form. Selection of papers and data-extraction were performed in duplicate by two researchers.

### Study population and design

To check the statistical assumptions and to compare the different statistical approaches, we used data of the HPV Amongst Vaccinated And Non-vaccinated Adolescents (HAVANA)-study. The study design of this observational cohort study has been described previously [12–14]. In brief, 29,162 girls born in 1993 or 1994 who were eligible for the catch-up campaign (three-doses of bivalent HPV vaccine) in 2009 and 2010 in the Netherlands were approached to participate in the study approximately one month before vaccination was offered. All participants provided written consent and the study was approved by the medical ethics committee (VU University Medical Center, Amsterdam). In total 1832 vaccinated and unvaccinated participants were included and asked to provide yearly follow-up with vaginal self-swabs and questionnaires. For current analyses, we included data up to eight years post-vaccination and study participants had to be negative for HPV16, 18, 31, 33 and 45 (vaccine and cross-protective types of the bivalent HPV vaccine) at baseline. Exposure was defined as having received the full recommended schedule of the bivalent HPV vaccine (three-doses at 0, 1 and 6 months) compared to unvaccinated women. Participants with an incomplete vaccination schedule were excluded from the analyses.

### HPV DNA detection and genotyping

HPV DNA testing was done by SPF_10_-LIPA_25_ system, with storage of vaginal self-swabs and methods used for HPV DNA detection and genotyping described in detail elsewhere [12, 15].

### Statistical analysis

We calculated the crude VE as 1 minus the hazard or rate ratio (*100%) using the different statistical approaches identified in the literature. Analyses were performed against a combined outcome of vaccine types HPV16/18 and cross-protective types HPV31/33/45. Persistence was defined on a type-specific level as being negative at baseline, followed by two consecutive positive rounds of testing. To be counted as a persistent case during follow-up, participants needed to have a persistent infection for at least one of these HPV types. In addition, at each time point a participant was evaluated to determine if they had a persistent infection based on previous time points. Person-time was counted from at least three consecutive rounds of participation, in order to be able to detect the endpoint of persistent infection based on three consecutive testing time points. Examples of calculating endpoints and person-time can be found in Additional file 2. Data analysis was performed using SAS 9.4 (SAS Institute Inc. 2010, USA).

## Results

### Literature search

**Fig. 1:**
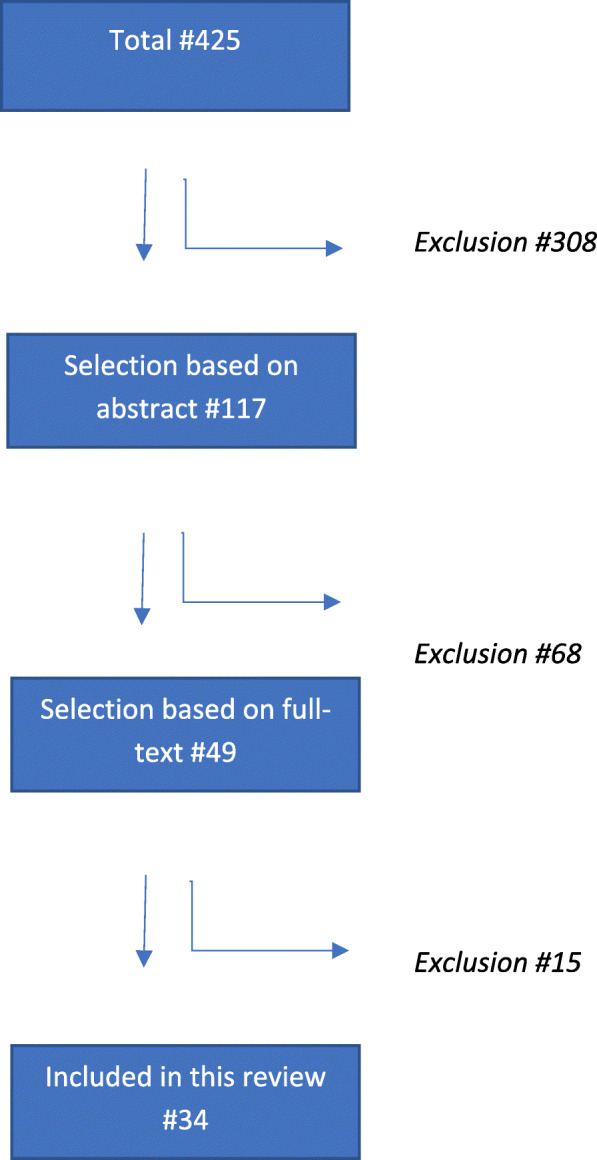
Flowchart of systematic literature search

The systematic literature search resulted in 425 articles, of which after selection (title and abstract) 49 remained for full text screening. Of these, four were excluded because of the wrong publication type (e.g. comment or review), seven because a lack of an actual vaccine effectiveness/efficacy calculation, and in four studies a different outcome other than the one of interest was reported, leaving a total of 34 articles (32 randomized controlled trials and 2 observational cohort studies) for inclusion [13, 14, 16–46]. (Fig. 1) This resulted in 35 analyses regarding vaccine efficacy/effectiveness of persistent HPV infections. Four different analysis methods were observed. Two methods provided an estimate of rate ratios either via Generalized Estimating Equations (GEE) using a Poisson model (*n* = 2), or via direct comparison of independent incidence rates using the Conditional exact method (*n* = 31) [47, 48], which assumes that the number of events from one group, given the total number of events in both groups, follows a binomial distribution under the null hypothesis using identical Poisson processes in the vaccinated and unvaccinated group [49]. The other two methods provided an estimate of hazard ratios either via the Cox proportional hazards model (*n* = 2), or via the Prentice Williams Peterson total time (PWP-TT) approach (*n* = 1). In all papers vaccine efficacy/effectiveness was calculated as 1 minus the rate ratio, or hazard ratio, times hundred percent. The PWP-TT is a survival method for recurrent events taking into account total time at risk, assuming event-specific hazards, in which the hazard is allowed to differ for a subsequent event [50, 51]. The GEE Poisson approach counts multiple events per participant (either over time or at the same time point) considering person-time. Only the first event is counted in both the conditional exact method using the binomial distribution and the Cox method. The Cox approach uses time until first event. Studies using the conditional exact method for comparing two independent Poisson rates using a binomial distribution varied in the denominator of outcome variable, either being total number of participants or number of person years observed (Table 1). An important assumption of Cox regression is that the hazard ratio is constant over time (proportional hazard assumption), while for the GEE Poisson and Conditional exact method, a constant rate of events over time would give the most stable estimates [52, 53]. The four methods vary in how they handle missing data. An overview of the different methods and their assumptions is shown in Table 2.

**Table 1:** Methods used to evaluate the VE against persistent infections and analyses from included studies

Type of study	Definition of persistence	Duration of persistent infection	VE analysis method	Calculation of infection rates
- Observational [13, 14] - Experimental [13, 16–40, 42–46]	- 2 consecutive measurements positive [13, 16–40, 42–46]. - 2 consecutive measurements: positive preceded by a negative measurement [14] - Sequence of positive measurements over a certain time span [18, 41]	- 6 months^a^ [17, 20, 22, 25, 26, 28–30, 34–39, 43, 44, 46] - 12 months^a^ [13, 14, 27, 40] - 6/12 months^a^ [16, 18, 19, 21, 23, 24, 31–33, 41, 42, 45]	- Conditional exact for comparing two independent Poisson rates using a binomial distribution (14,15,24–28 [39, 41–43] ,16, 35–44,17–23) - GEE Poisson [13, 40] - Cox Proportional Hazard [22, 38] - Prentice Williams Peterson total time approach [14]	- Number of cases/number of participants [17–19, 21, 22, 25, 27, 31–33, 38, 41–44] - Number of cases/person years at risk [13, 14, 16, 20, 23, 24, 26, 28–30, 34–37, 39, 40, 45, 46]

* *Although it was stated as 6- or 12-month persistent infections authors specified durations varying between at least 4 to 6 months or 10 to 12 months respectively*

**Table 2:** Analysis methods for vaccine effectiveness against persistent HPV infections

	Conditional exact method for comparing two independent Poisson rates using a binomial distribution	Cox proportional hazard	GEE Poisson	Prentice Williams Peterson-Total time
Outcome	Rate ratio	Hazard ratio	Rate ratio	Hazard ratio
Assumption(s)	* Rate of events constant over time * Groups are considered to be equally exposed [52]	* Proportional hazard assumption (hazard ratio over time should be constant) * Independence assumption (estimate only for 1st event) [52]	* Rate of events constant over time [52] * Measurements are independent across subjects * Measurements may be correlated within subjects	* Event specific baseline hazard (baseline hazard for k^th^ event allowed to be different) [51]
Check assumptions in HAVANA	Assumption for constant rate over time violated among unvaccinated	Proportional hazard assumption not violated	Assumption for constant rate over time violated among unvaccinated	Assumption for event-specific hazard not violated

### Assumptions

**Fig. 2:**
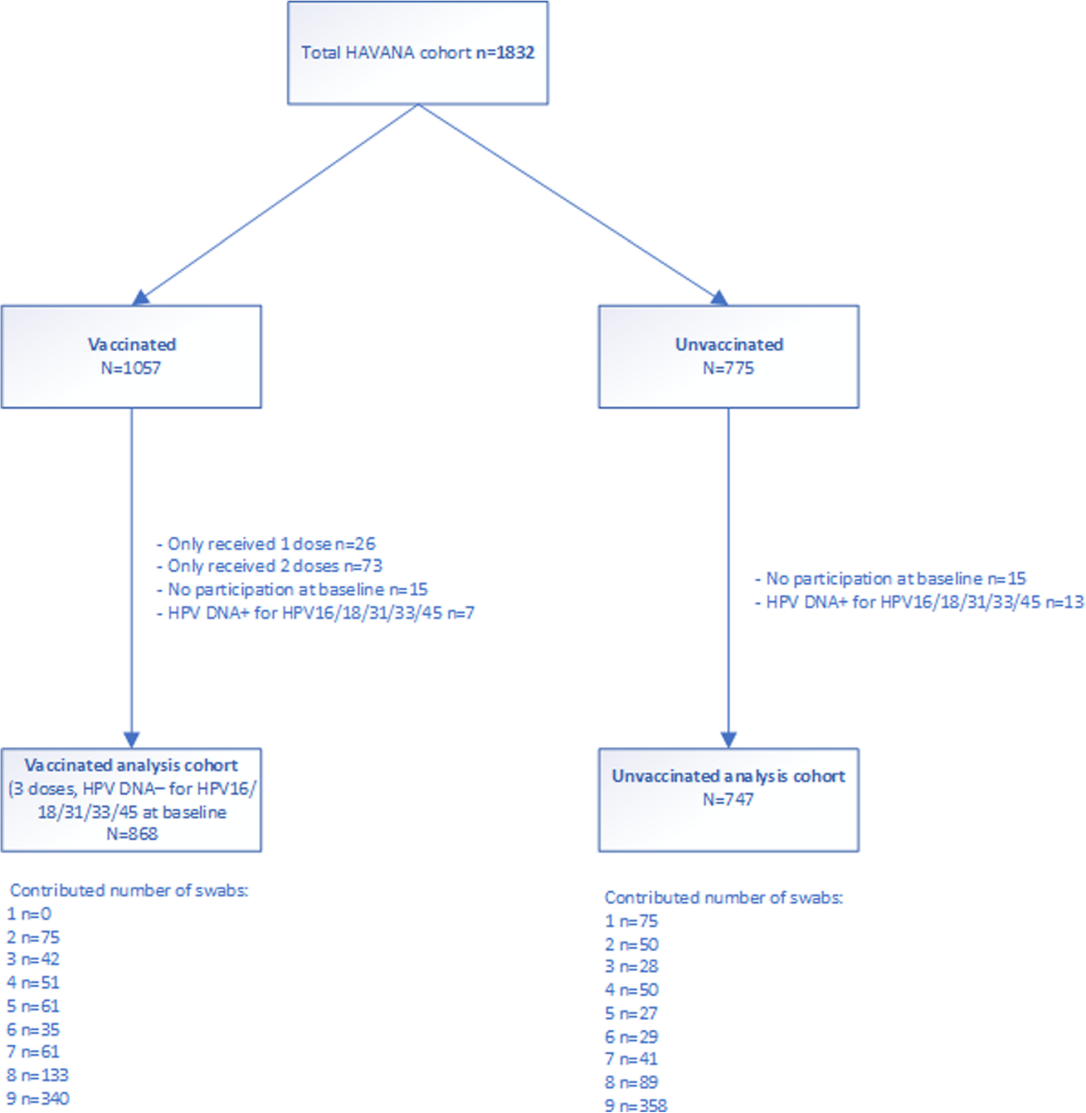
Flowchart of analysis population

Data from the observational HAVANA-study [12–14] were used to check the assumptions and to calculate the VE estimates using the different methods. In total, 1615 participants were included in the current analyses. These participants provided a baseline sample and were negative at baseline for HPV16/18/31/33/45. Of these, 747 were unvaccinated and 868 were fully vaccinated (three doses at 0, 1 and 6 months), where vaccination occurred approximately one month after inclusion into the study. (Fig. 2) We checked whether assumptions regarding constancy of the hazard ratio, constancy in the rate of events and the event-specific hazard assumption hold in the HAVANA-study for persistent HPV16/18 and HPV31/33/45 infections. To check the proportional hazard assumption (Cox regression), we added the interaction between vaccination status and time to the Cox model. Based on the interaction term between vaccination and time, the proportional hazard assumption was not violated for both vaccine (*p* = 0.19) and cross-protective types (*p* = 0.60). To check for a constant event rate (GEE Poisson and Conditional exact method), we modeled the persistence rate as a function over time stratified for unvaccinated and vaccinated participants. An increasing persistence rate (*p* < 0.01) for both vaccine types and cross-protective types over time was observed among unvaccinated, but not among vaccinated participants (*p* = 0.14 and *p* = 0.17 respectively). This indicates that the assumption of constant event rate was violated. (Table 3). In order to check whether there is an event specific hazard (PWP-TT), we estimated the persistence rate for each subsequent event number. The hazard for subsequent infections indeed seems to be different, as the persistence rate (PR) for a subsequent persistent infection in the total population was higher for the second infection compared to the first infection. The PR ratio (PRR) for the second infection was 7.38 95%CI 2.95–18.45 for HPV16/18 and 5.95 (95%CI 1.85–19.09) for HPV31/33/45 compared to the first infection (Table 4).

**Table 3:** Persistence rates (PR) and persistence rate ratios (PRR) for HPV16/18 and HPV31/33/45 (vaccine and cross-protective types) over time, in years since vaccination

*Yrs. Since vaccination*	*Vaccination status*	*N*	*Vaccine types (HPV16/18)*			*Cross-protective types (HPV31/33/45)*		
			*# infections*	*PR per 100 PY (95%CI)*	*PRR (95%CI)*	*# infections*	*PR per 100 PY (95%CI)*	*PRR (95%CI)*
*2*	Unvaccinated	551	2	0.18 (0.05–0.73)		2	0.18 (0.05–0.73)	Ref
	Vaccinated	626	0	0.00 (0.00–0.59)		1	0.08 (0.01–0.57)	0.44 (0.04–4.85)
*3*	Unvaccinated	513	2	0.19 (0.05–0.78)		2	0.19 (0.05–0.78)	Ref
	Vaccinated	567	0	0.00 (0.00–0.65)		2	0.18 (0.04–0.71)	0.90 (0.13–6.42)
*4*	Unvaccinated	472	7	0.74 (0.35–1.56)		4	0.42 (0.16–1.13)	Ref
	Vaccinated	515	0	0.00 (0.00–0.72)		3	0.29 (0.09–0.90)	0.69 (0.15–3.074)
*5*	Unvaccinated	455	10	1.10 (0.59–2.04)		3	0.33 (0.11–1.02)	Ref
	Vaccinated	472	0	0.00 (0.00–0.78)		1	0.11 (0.01–0.75)	0.32 (0.03–3.09)
*6*	Unvaccinated	447	18	2.01 (1.27–3.20)	Ref	12	1.34 (0.76–2.36)	Ref
	Vaccinated	438	1	0.11 (0.02–0.81)	0.06 (0.01–0.42)	2	0.23 (0.06–0.91)	0.17 (0.04–0.76)
*7*	Unvaccinated	433	11	1.27 (0.70–2.99)	Ref	5	0.58 (0.24–1.39)	
	Vaccinated	448	2	0.22 (0.06–0.89)	0.18 (0.04–0.79)	0	0.00 (0.00–0.82)	
*8*	Unvaccinated	414	6	0.72 (0.33–1.61)		9	1.09 (0.57–2.09)	Ref
	Vaccinated	429	0	0.00 (0.00–0.86)		5	0.58 (0.24–1.40)	0.54 (0.18–0.60)

PR = persistence rate (with 95%CI), PRR = persistence rate ratio (with 95%CI), py = person years, Yrs = years

* Trend in persistence rate over time for HPV16/18 among unvaccinated, p < 0.01, among vaccinated p = 0.14

** Trend in persistence rate over time for HPV31/33/45 among unvaccinated, *p* < 0.01, among vaccinated p = 0.17

**Table 4:** Persistence rates per event (event 1 is the first persistent infection with a vaccine /cross-protective type, event 2 is the second persistent infection, with at least one negative observation in between type-specific persistent infections, event 3 is the third persistent infection, with at least one negative observation in between type-specific infections)

*Vaccination Status*	*Event*	*Cases*	*Person time*	*PR per 100 PY*	PRR per 100 PY
*Vaccine types (HPV16/18)*					
Unvaccinated	1	51	3792	1.35 (1.02–1.77)	Ref
	2	5	95	5.26 (2.19–2.64)	3.91 (1.56–9.80)
	3	0	17		
Vaccinated	1	3	4100	0.07 (0.02–0.23)	
	2	0	4		
	3	0	0		
* Cross-protective types (HPV31/33/45)*					
Unvaccinated	1	34	3813	0.89 (0.64–1.25)	Ref
	2	3	60	5.00 (1.61–15.50)	5.61 (1.72–18.26)
	3	0	6		
Vaccinated	1	14	4082	0.34 (0.20–0.58)	
	2	0	60		
	3	0	0		

PR = persistence rate, PRR = persistence rate ratio, py = person years

### Vaccine effectiveness

We used the different methods found through the systematic literature search to calculate the VE against persistent infections (with an interval of at least twelve months) with vaccine or cross-protective types up to eight years post vaccination in the HAVANA-study. Definitions used for the analyses are shown in Table 5, with examples of calculations in Additional file 2. To estimate VE for the conditional exact method using a binomial distribution, whether a participant had a persistent infection during follow-up was used as the outcome (persistent case), assuming that the number of cases in each of the arms are independent Poisson random variables. For the PWP-TT participants with multiple simultaneous persistent infections, individuals were counted as having one persistent event at that specific time point. While in the GEE Poisson approach, all simultaneous infections for different HPV types were counted and all subsequent events were counted as multiple events. For the Cox PH analysis only the first infection was used.

**Table 5:** Definitions and analysis of cases and time at risk

*Analysis* *Method*	*Case definition*	*Person-time definition*
Conditional exact method for comparing two independent Poisson rates using a binomial distribution	Two consecutive measurements positive for the same HPV type. The participant is counted as a case if one or more persistent infections occur.	Data for two consecutive rounds counts as 1 person-year, each additional consecutive round adds another person-year. After a missing data point counting continues. Counting stops after event or at the end of follow-up.
Cox PH	Two consecutive measurements positive for the same HPV type. The participant is counted as a case if one or more persistent infections occur.	Data for two consecutive rounds counts as 1 person-year, each additional consecutive round adds another person-year. Person time is censored at event, loss to follow-up or end of follow-up; half-time censoring was applied.
GEE Poisson	Two consecutive measurements positive for the same HPV type. Multiple events can occur within one participant. In our study to be counted as next infection after at least one negative round was observed. The number of infections is counted.	Data for two consecutive rounds counts as 1 person-year, each additional consecutive round adds another person-year. After a missing data point counting continues. Counting stops at the end of follow-up.
PWP-TT	Two consecutive measurements positive for the same HPV type. Multiple events can occur within one participant, in our study to be counted as next infection; at least one negative round should be observed. The number of infections is counted. Analyses are stratified for sequential events.	Data for two consecutive rounds counts as 1 person-year. After a missing data point counting continues. Counting stops at the end of follow-up.

**Fig. 3:**
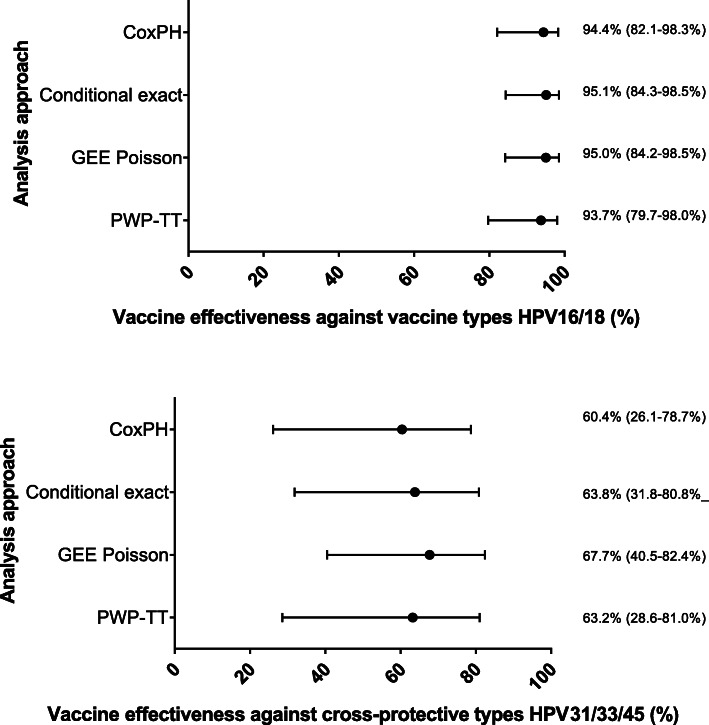
Crude vaccine effectiveness up to eight years post-vaccination against persistent HPV16/18 and HPV31/33/45 infections observed in the HAVANA-study using different statistical approaches

Through the model assumption checking, we found that the Cox model and the PWP-TT method were the only approaches for which the statistical assumptions were not violated using the HAVANA-study data. The PWP-TT takes into account the possibility of multiple infections during the follow-up time. Whereas the Cox model can only account for one event when using a pooled outcome of vaccine types or cross-protective types and multiple type infections occurring at the same moment. The estimated VE for vaccine types using the PWP-TT method was 93.7% (95%CI 79.7–98.0%) and for cross-protective types the VE was 63.2% (95%CI 28.6–81.0%). Despite observing small differences in estimates and confidence intervals with the other methodological approaches, the obtained VE estimates and corresponding 95%CI using any of the methods overlapped with the estimates obtained using the PWP-TT method. The VE against persistent HPV16/18 infections measured by the different methods varied between 93.7 and 95.1%, and for HPV31/33/45 between 60.4 and 67.7%, with the lowest point estimates given by the two methods for which the model assumptions were not violated. (Fig. 3).

## Discussion

### Main findings

Our literature search identified four approaches for calculating the vaccine efficacy/effectiveness against persistent HPV infections. These different methods vary in their underlying assumptions and measures. Based on our observational study, the Cox Proportional hazard and PWP-TT method were the only ones whose assumptions were not violated in our observational cohort study data. In addition, the PWP-TT has the advantage that it uses information from the complete follow-up time, compared to a single event time used in the Cox model. Compared to the PWP-TT, the VE estimates against HPV16/18 and HPV31/33/45 calculated by the other methods were quite comparable, and seemed robust to violations of the underlying assumptions.

### Statistical approaches

The four different methods found in our search vary in their underlying assumptions, but also in how they handle missing observations or loss-to-follow up. In our systematic search for methods to analyze VE against persistent HPV infections, we found both randomized controlled trials and observational studies. An important difference is that in randomized controlled trials there is no confounding, while in observational studies, adjustment for confounding is needed.

### Assumptions

Using data from an observational cohort study we checked whether the assumptions of the various methods hold. The proportional hazard assumption for Cox models was not violated in our data. However, as follow-up time increases, the proportional hazard between vaccinated and unvaccinated might vary over time, for example, if vaccine protection might wane or gets boosted by exposure to the virus [53]. Malagon et al. suggested waning of HPV-cross-protection after five years post-vaccination [4]. However, recent studies did not show indications for waning of cross-protection [14, 54–56]. In our data, the assumption with regard to constancy of the event rate was violated in unvaccinated participants, which was to be expected based on existing literature about HPV prevalence over time. For example, Lenselink et al. have shown an increase in HPV prevalence till 22 years of age [9]. We also checked whether we found an event-specific hazard for subsequent infections.

In our study, observed follow-up for a second and third infection among vaccinated was small, hence interpretation of the findings in this group is difficult. Among unvaccinated, we clearly observed a higher rate of events amongst those who already had an event. In the literature so far, no clear consensus regarding the risk for a new infection after a previous infection has been reached [8, 57–60].

As analyses using PWP-TT are stratified by event number, and slightly wider confidence intervals are estimated, therefore the event-specific estimates could become unreliable if there are a limited number of events in a stratum [50].

A problem that might arise when using GEE Poisson models to estimate the VE is an excess of zero counts when the vaccine is highly effective, which leads to overdispersion. In the presence of overdispersion, the variance of the parameters within the model will be underestimated [61]. Based on the negative dispersion parameter [62], it seems that the observed variance within the data was higher than what was expected under the GEE Poisson model. However, estimating the VE using a negative binomial model showed comparable VE estimates, 95.0% (95%CI 84.1–98.5%) against vaccine types and 67.5% (38.2–82.9%) against cross-protective types, which may suggest robustness of the estimates despite the presence of overdispersion.

### Vaccine effectiveness estimates

The obtained estimates from all the methods where assumptions were violated were quite comparable to the CoxPH and the PWP-TT methods, for which assumptions were not violated in our observational study. In addition, we observed comparable, or slightly higher point estimates, for the observed vaccine effectiveness against vaccine and cross-protective HPV-types in comparison to previous studies evaluating vaccine effectiveness against persistent infections after vaccination with the bivalent HPV vaccine in HPV naïve women [16, 24, 45, 63].

We did not find evidence that the vaccine effectiveness estimates were influenced due to a violation of the underlying model assumptions. However, as follow-up time and the number of persistent infections increases, significant differences between methods might develop. Difference between methods of calculating VE may also occur when these methods are applied in study populations at higher risk for HPV infections.

Although we found comparable estimates using different methods, we suggest the PWP-TT as a valid and preferable method to estimate the VE against persistent HPV infections in observational studies. This recommendation is based our findings regarding the violation of the model assumptions with respect to constant rates or ratios and common baseline hazard, combined with available literature, and our comparison analysis from complete follow-up data to calculate VE against persistent HPV infections in observational studies.

For our analyses, we used a combined endpoint of vaccine and cross-protective types to estimate the VE. An alternative for using combined endpoints would be measuring type-specific VEs and pooling these. A limitation of the PWP-TT method when using a combined endpoint for multiple HPV types is that simultaneous infections cannot be counted separately, while infections for different types later in time are counted as separate events. However, running type-specific vaccine effectiveness models will overcome this potential limitation.

## Conclusion

For the four methods used to calculate VE in our observational study, the estimates were comparable between those that did not violated statistical assumptions, the CoxPH and the PWP-TT methods, and those that did violate assumptions, GEE using a Poisson and conditional exact methods.

For monitoring the effectiveness of HPV vaccination in cohorts of young adolescents/adults with increasing HPV prevalence the PWP-TT approach seems is recommended as valid and preferable, as it considers the varying rates of events and uses data of the whole follow-up period. A limitation when using this method might occur when using combined endpoints for multiple HPV types, since this cannot be taken into account in the model. Further studies should focus on populations with higher HPV persistence rates in order to confirm our findings.

## Supplementary information

**Additional file 1.** Search query

**Additional file 2.** Examples of calculations for different approaches with regard to number of events and person time at risk.

## Data Availability

The datasets used and/or analysed during the current study are available from the corresponding author on reasonable request.

## Abbreviations

CE: Conditional Exact
Cox PH: Cox Proportional Hazard
GEE: Generalized Estimating Equations
HAVANA: HPV Amongst Vaccinated And Non-vaccinated Adolescents
HPV: Human papillomavirus
hrHPV: High-risk HPV
inf: Infections
PR: Persistence rate
PRR: Persistence rate ratio
PWP-TT: Prentice Williams and Peterson Total-Time
Py: Person-years
VE: Vaccine Effectiveness
WHO: World Health Organization
Yrs: Years
