# Supplementary material for: Measuring vaccine effectiveness against persistent HPV infections: a comparison of different statistical approaches
Source: BMC Infect Dis. 2020 Jul 8;20:482. doi: 10.1186/s12879-020-05083-7 (PMC7341660; doi:10.1186/s12879-020-05083-7)
Supplement: Supplementary file 1 — Additional file 1. Search query [file 12879_2020_5083_MOESM1_ESM.docx]

***Additional file 1: Search query***

*Search criteria for the Systematic literature search regarding vaccine effectiveness/efficacy against persistent HPV infections.*

| #1 | Search **papillomavir*[tiab] or hpv[tiab] or hpv*[tiab] or papilloma*[tiab] or papillomaviridae[mh]** | #73441 |
| --- | --- | --- |
| #2 | Search **vaccin*[tiab] or immunisat*[tiab] or immunizat*[tiab] or vaccines[mh] or vaccination[mh] or immunization[mh:noexp] or papillomavirus vaccines[mh]** | 387929 |
| #3 | Search **persist*[tiab]** | 446196 |
| #4 | Search **effectiv*[tiab] or effica*[tiab]** | 387929 |
| #5 | #1 AND #2 AND #3 AND #4 | 425 |
