# Supplementary material for: Measuring vaccine effectiveness against persistent HPV infections: a comparison of different statistical approaches
Source: BMC Infect Dis. 2020 Jul 8;20:482. doi: 10.1186/s12879-020-05083-7 (PMC7341660; doi:10.1186/s12879-020-05083-7)
Supplement: Supplementary file 2 — Additional file 2. Examples of calculations for different approaches with regard to number of events and person time at risk. [file 12879_2020_5083_MOESM2_ESM.docx]

***Additional file 2: Examples of calculations for different approaches with regard to number of events and person time at risk***

Only participants negative for HPV16/18/31/33/45 at baseline were included for these analyses.

| Case nr. | Prevalence | | | | | | | | | Number of events | | | | Person-time at risk | | | |
| --- | --- | --- | --- | --- | --- | --- | --- | --- | --- | --- | --- | --- | --- | --- | --- | --- | --- |
|  | R0 | R1 | R2 | R3 | R4 | R5 | R6 | R7 | R8 | CE | CoxPH | GEE Poisson | PWP-TT | CE | CoxPH | GEE Poisson | PWP-TT |
| 1 | NEG | POS | POS | NEG | POS | POS | NEG | NEG | NEG | 1 case | 1 case | 2 inf | 2 inf | 2 yr | 1.5 yrs | 8 yrs | 8 yrs |
| 2 | NEG | NEG | NEG | POS | POS | NEG | NEG | NEG | NEG | 1 case | 1 case | 1 inf | 1 inf | 4 yrs | 3.5 yrs | 8 yrs | 8 yrs |
| 3 | NEG | NEG | . | NEG | NEG | NEG | NEG | POS | POS | 1 case | 0 case | 1 inf | 1 inf | 5 yrs | 0 yrs | 5 yrs | 5 yrs |
| 4 | NEG | NEG | NEG | . | NEG | NEG | NEG | NEG | NEG | 0 case | 0 case | 0 inf | 0 inf | 6 yrs | 2.5 yrs | 6 yrs | 6 yrs |

CE= conditional exact method for comparing two independent Poisson rates using a binomial distribution, Inf=Infections, PWP-TT= Prentice Williams Peterson-Total time, Yr(s)=year(s)
